# Virtual reality therapy: A promising solution to chronic pain management amidst an opioid crisis

**DOI:** 10.7189/jogh.13.03033

**Published:** 2023-06-23

**Authors:** Huda Ahmed, Hasan Mushahid, Muhammad Hamza Shuja

**Affiliations:** Department of Medicine, Dow University of Health Sciences, Baba e urdu road, Saddar, Karachi, Pakistan

**Figure:**
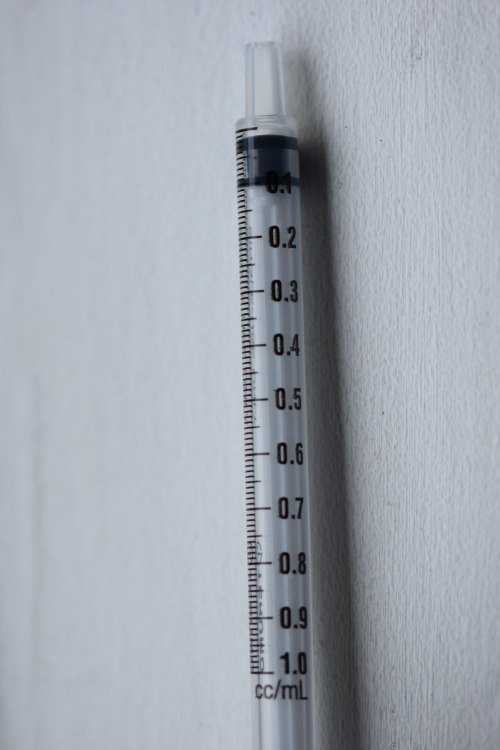
Photo: The ongoing opioid crisis. Source: https://flic.kr/p/QADqPT. Free to use under CC0 1.0 Universal license.

## CHRONIC PAIN AND ITS TREATMENTS

Chronic pain can be characterised as the body’s alarm malfunctioning, leading to spontaneous pain, allodynia, and hyperalgesia, often due to an injury to the central and peripheral nervous systems’ nociceptive circuitry, rendering the dorsal horn of the spinal cord more excitative [1]. Clinically, it is defined as pain that lasts more than three months and/or beyond the expected healing time [2]. Recent data reveal that 20.4% of the world's population is affected by chronic pain, highlighting the need for effective management strategies [1,3].

Current guidelines for chronic pain management suggest an interdisciplinary approach that considers the biological, psychological, and social factors underlying chronic pain [3]. As of 2022, the Centers for Disease Control and Prevention (CDC) guidelines have established nonpharmacological therapies, such as physiotherapy, exercise, and cognitive behavioural therapy, as the preferred treatment for chronic pain [4]. Non-opioid analgesics, such as paracetamol and non-steroidal anti-inflammatory drugs (NSAIDs), are recommended as second-line treatment, but their use increases the risk of gastrointestinal bleeding [4,5]. Both the CDC and World Health Organization (WHO) guidelines (summarised in **Table 1**) strongly emphasise that opioids should be the last resort for chronic pain relief, only considered after nonpharmacological therapies and non-opioid analgesics have failed, except in patients receiving cancer treatment or hospice care [4].

**Table 1:** Comparison of available treatment options for chronic pain: A summary of guidelines by WHO and CDC

Treatment options	Mechanism of action	Advantages	Disadvantages	Guidelines
Non-opioid analgesics	Inhibit COX-1 and COX-2	Reduces inflammation, relieves pain.	Stomach discomfort, stomach bleeding, GI issues.	WHO guidelines recommend non-opioid analgesics as first-line therapy. CDC guidelines recommend nonpharmacological therapies such as physiotherapy, exercise, and cognitive-behavioural therapy as the preferred first-line treatment over non-opioid analgesics.
Mild opioids (codeine, dihydrocodeine)	Target μ-opioid receptor	Effective in pain management when non-opioid analgesics fail.	Can cause dependence and tolerance, addiction, and opioid use disorder.	WHO guidelines recommend mild opioids as the second-line therapy if non-opioid analgesics are ineffective. CDC guidelines prioritise nonpharmacological therapy over opioid use.
Strong opioids (morphine)	Target μ-opioid receptor	Can effectively relieve musculoskeletal and neuropathic pain in the short term due to the rapid onset of action.	Can cause dependence and tolerance, addiction, and opioid use disorder.	WHO guidelines recommend using strong opioids as the last resort when non-opioid and mild opioid therapies have failed. CDC guidelines prefer non-pharmacological therapy, with opioids prescribed only after the failure of the initial two treatment options, except for patients in cancer treatment or hospice care.
VR therapy	Rely on the concept of modulating neuroplasticity	Non-invasive, few side effects, can be implemented remotely.	Requires an initial costly investment, may not be suitable for all patients.	An emerging therapeutic option which gained acknowledgement from WHO and CDC.

COX-1 – cyclooxygenase, WHO – World Health Organization, CDC – Centers for Disease Control and Prevention, GI – gastrointestinal, VR – virtual therapy

### Drawbacks associated with the use of opioids

Despite recommendations from the CDC and WHO, opioids have long been considered a valuable option for managing chronic pain due to their ability to target the μ-opioid receptor, which reduces nociceptive excitation and impedes synapses, making them more effective analgesics as compared to other methods [6]. Despite this greater efficacy, they have some drawbacks, as the prolonged use of opioids can result in tolerance and dependence in all ages, genders, and ethnicities. Individuals can become physically and psychologically reliant on opioids to manage pain or experience euphoria, possibly leading to addiction in approximately 8-12% of cases [7]. Research has shown that addiction to opioids is primarily the result of damage to the amygdala, leading to irregular reward-stress functions and hyperkatifeia, a state characterized by heightened negative emotions during withdrawal that perpetuates the addiction cycle [6]. Consequently, chronic pain patients have an even greater frequency of substance addiction disorders than the general population (8.1%) [8]. Despite advancements in treatment regimens and increasing awareness of opioid-related adversities, opioid related-deaths have increased by 71% since 2013. In 2019, an estimated 50 million individuals worldwide were living with opioid use disorder, creating a substantial economic burden of US$17.8 billion in healthcare expenses annually in the US alone [7].

The current opioid crisis demands innovative solutions to effectively combat addiction. Despite being one of the few available options, physician-supervised tapering has demonstrated limited success with only a 36.4% success rate after a six-month follow-up [9]. Patients often experience relapses due to inadequate management of withdrawal symptoms during tapering, necessitating improved withdrawal management protocols [10]. While harm reduction programmes like syringe exchange programmes have increased access to naloxone (an FDA-approved drug that reverses the effects of opioid overdose), their impact on opioid addiction remains indirect [11]. Counselling services and controlled dispensing of opioids have also been explored, but their effectiveness in managing addiction behaviours has been insufficient [11]. Novel approaches for addressing the underlying causes of addiction must be developed to mitigate the escalating economic and social costs of the opioid crisis.

### Virtual reality-based therapy as an emerging technology in treating chronic pain

Given the limitations of opioids, virtual reality (VR)-based therapy has shown promise as an effective, accessible, and low-cost treatment for chronic pain management. It involves the use of advanced equipment, including a headset, to create a virtual environment that immerses patients in an interactive and engaging experience aimed at reducing pain and promoting relaxation [12]. The potential of VR therapy to revolutionise chronic pain management has gained increasing attention recently, with growing evidence of its effectiveness and potential for widespread implementation.

Multiple studies have demonstrated the efficacy of VR therapy in reducing chronic pain. A randomised control trial of 30 participants by Pekyavas et al. [13] found that, when compared to home workout regimens, VR gaming (including boxing, bowling, and tennis) significantly reduced scores for chronic pain patients with subacromial impingement syndrome (*P* < 0.005) [13]. These findings suggest that introducing VR therapy into existing physiotherapy programmes may have significant benefits, and are one of the first observations to assess VR therapy’s impact on musculoskeletal pain problems that could open new horizons for its implementation [13]. For long-term assessments, Botella et al. [14] conducted a pilot study of six female fibromyalgia (FM) patients and found that 10 two-hour VR sessions significantly improved scores on the FM Impact Questionnaire when performed over six months. They also reported high rates of patient satisfaction, as they were able to picture VR scenarios to relax after the sessions had ended. These patients also preferred the accessibility of VR, as they could receive effective pain relief at home [14]. Additionally, a meta-analysis of 31 studies by Huang et al. [15] reported a reduction in anxiety, discomfort, and mental stress of chronic pain compared to standard treatment using opioids (*P* < 0.001). These psychological benefits are essential in overcoming opioid addiction, as withdrawal symptoms often include anxiety and restlessness, leading to relapse [10].

While the exact mechanisms underlying the analgesic effect of VR therapy are not yet fully understood, it is hypothesised that the immersive experience provided by VR distracts patients from their pain. Another approach to VR therapy involves teaching patients new pain management techniques which can enhance their emotional regulation abilities and reduce reliance on opioids [12]. By equipping patients with new, personalised coping strategies, VR therapy can empower patients to manage their pain more effectively without the need for opioids, which is especially relevant given the ongoing opioid crisis. As such, it holds immense promise as a non-invasive and effective tool for managing chronic pain.

The use of VR therapy shows numerous advantages over some of the conventional modes of treatment. As more customer-focused VR devices become available, fewer in-person visits to healthcare facilities may be necessary if patients are able to use VR therapy at home. Likewise, it can be highly individualised based on a patient’s unique demands. For instance, virtual settings and scenarios can be adapted to a patient’s preferences and pain treatment objectives. Furthermore, the therapy is non-invasive and does not involve any medical procedures or prescription drugs, so individuals who might not be tolerant of other forms of invasive procedures may opt for it as a safer alternative. Besides this, it has demonstrated few side effects, with existing ones being frequently minor and transient, such as light-headedness or motion nausea. Lastly, VR therapy has been shown as an effective substitute for opioids, which have a variety of unfavourable side effects and can cause addiction. These benefits favour the use of VR therapy for treatment of chronic pain.

## CONCLUSION

VR therapy could potentially transform current chronic pain management. Its scalability, accessibility, and potential for remote therapy options make it a cost-effective alternative to opioids. However, the current evidence supporting its efficacy is limited by small sample sizes, a lack of a standardised protocol, and variations in outcome measures. These factors make it difficult to quantify the exact benefits that VR therapy may provide for more diverse patient populations. Further large-scale investigations with sub-group analyses to identify heterogeneities are necessary to fully establish its long-term effects and underlying biological mechanisms. By gaining a deeper understanding of these mechanisms, researchers can develop more targeted and effective VR therapies for chronic pain management, ultimately reducing opioid-related adversities and improving patients' quality of life in an ongoing opioid crisis.
